# Vertigo Induced During Coitus

**DOI:** 10.3389/fneur.2018.01187

**Published:** 2019-01-11

**Authors:** Sun-Uk Lee, Hyo-Jung Kim, Ja-Won Koo, Jeong-Yoon Choi, Ji-Soo Kim

**Affiliations:** ^1^Department of Neurology, Seoul National University College of Medicine, Seoul, South Korea; ^2^Dizziness Center, Clinical Neuroscience Center, Department of Neurology, Seoul National University Bundang Hospital, Seongnam, South Korea; ^3^Research Administration Team, Seoul National University Bundang Hospital, Seongnam, South Korea; ^4^Department of Otolaryngology-Head and Neck Surgery, Seoul National University College of Medicine, Seoul National University Bundang Hospital, Seongnam, South Korea

**Keywords:** vertigo, nystagmus, coitus, Meniere's disease, superior canal dehiscence, high jugular bulb anomaly

## Abstract

**Objectives:** The aim of this study was to describe the clinical features of vertigo developed during sexual intercourse.

**Methods:** We retrospectively reviewed the clinical and laboratory findings of seven patients who reported recurrent vertigo during sexual intercourse.

**Results:** All the patients reported spinning sensation for a few minutes to 1 h, which developed during the coitus. Most patients (6/7, 86%) reported associated auditory symptoms including tinnitus (*n* = 4), ear fullness (*n* = 2), autophony (*n* = 1), hearing impairment (*n* = 1), or hyperacusis (*n* = 1). Four patients reported the vertigo to occur exclusively during sexual intercourse or masturbation while the other three patients also experienced vertigo during other physical activities. Underlying disorders included Meniere's disease (*n* = 3), superior canal dehiscence (*n* = 1), and high jugular bulb anomaly (*n* = 1) while the remaining two patients had no identifiable causes.

**Conclusions:** Various disorders may cause coital vertigo probably due to disruption of the mechanism that normally refrains the increased intracranial pressure from being directly transferred to the peripheral vestibular organs.

## Introduction

Like other physical activities, sexual intercourse increases the pulse rate, respiration rate and blood pressure. Furthermore, heart rate rises up to 110–180 beats per minute and respiration rate up to 40 breaths per minute during orgasm (1). These systemic and cerebral hemodynamic changes may explain the neurological disorders that occur in association with coitus, which include coital cephalalgia (2), transient monocular blindness (3), reversible cerebral vasoconstriction syndrome (4), transient global amnesia (5), syncope (6), subarachnoid and intracerebral hemorrhage (7, 8), and ischemic strokes (9).

Previously, transient vertigo has been reported during sexual intercourse after a stapes surgery (10), and has been ascribed to leakage of the periplymph due to increased intracranial pressure (ICP) during the coitus. However, the characteristics, underlying disorders, and mechanisms of coital dizziness/vertigo have not been explored systematically in a large number of patients. The aim of this study is to draw attention of physicians to this potentially important, but easily overlooked phenomenon.

## Methods

### Subjects

We reviewed seven patients (four women, age range: 41–68 years, mean age ± *SD* = 54 ± 10) who reported vertigo that had developed during sexual intercourse at the Dizziness Clinic of Seoul National University Bundang Hospital from 2003 to 2018.

### Bedside Neurotologic Examinations

All patients had evaluation of spontaneous, gaze-evoked nystagmus (GEN), bedside head-impulse tests (HITs) during visual fixation. Spontaneous and head-shaking nystagmus (HSN) were also observed on a monitor without visual fixation in darkness using video-Frenzel goggles (Easyeye, SLMED, Seoul, South Korea) (11, 12).

### Video-Oculography

Nystagmus was recorded binocularly at a sampling rate of 60 Hz using a video-oculography (SMI, Teltow, Germany) (13). Digitized eye position data were analyzed using MATLAB^®^ software. While wearing the video-oculography goggles in a seated position, spontaneous nystagmus was recorded both with and without fixation in the straight-ahead gaze. Also, GEN was recorded in the horizontal (±30°) and vertical (±20°) planes. HSN was induced by a passive head-shaking: The examiner pitched the patient's head forward by 30° to bring the horizontal canals (HCs) into the plane of stimulation. The patients' head was then grasped firmly with both hands, and shaken horizontally in a sinusoidal fashion at a rate of 2.8 Hz paced to the sound of a metronome for 15 s (13). Vibratory stimuli were applied to either mastoid and brow for 10 s with an interval of 5 s without visual fixation using VVIB 100 (Synapsys, Marseille, France). The frequency of the vibration was 100 Hz (±5%) and the contact area was 0.9 cm^2^. If the intensity of vibration-induced nystagmus (VIN) was different among the stimulation sites, the strongest one was adopted for analyses (14). Positional nystagmus was observed during serial changes of the positions, which included lying down, turning the head to either side while supine, straight head hanging, and Dix-Hallpike maneuver in each direction (12). Nystagmus was also recorded after hyperventilation for ~30 s while seated in darkness, taking an average of one deep breath per second (15). For evaluation of the Tullio phenomenon, 500 Hz and 1 kHz short tone bursts (stimulation rates: 1.1/s, 2.1/s, 3.1/s; rise and fall time: 2 ms; plateau time: 3 ms; intensity: 93 dB nHL) were used (16). Fistula test was performed by applying a positive-pressure insufflation to the ear canal with a Politzer bag. The nystagmus was compared before and after the pressure-insufflation for 30 s. A patient with superior canal dehiscence (SCD, patient 4) had a recording of HITs using a video-based equipment (SLMED, Seoul, South Korea).

### Other Neurotologic Evaluation

Patients also underwent measurements of bithermal caloric tests, and cervical and ocular vestibular-evoked myogenic potentials (VEMPs) with an evaluation of the threshold for cervical VEMPs. Detailed methods of each test have been described previously (13, 16–20).

All experiments followed the tenets of the Declaration of Helsinki and this study was approved by Institutional Review Board of Seoul National University Bundang Hospital (B-1506-302-109).

## Results

### Case Description

#### Patient 1

A 65~70-year-old woman reported transient vertigo for 6 years, which had become more frequent up to 2–3 times a month after initiation of estrogen treatment for breast cancer 2 years before. The vertigo had lasted about 30 min and accompanied ear fullness and hearing difficulty on the left ear. Her spouse reported that her vertigo frequently had occurred during sexual intercourse. Interictal examination showed no spontaneous or triggered nystagmus during positional maneuvers or after horizontal head-shaking. Pure tone audiometry (PTA) showed fluctuating left sensorineural hearing loss, especially in the lower frequency range. The results of bithermal caloric tests, cervical and ocular VEMPs, and brain MRIs and MR angiography were normal. She was diagnosed with Meniere's disease (MD), and the vertigo spell markedly decreased with medication including 12 mg of betahistine, 25/25 mg of spironolactone/hydrochlorothiazide per day.

#### Patient 2

A 55~60-year-old man presented recurrent vertigo and tinnitus for 3 years. The patient had been taking silodosin 4 mg a day for benign prostate hyperplasia. The vertigo lasted about 30 min, and accompanied nausea, vomiting, and tinnitus in the right ear. The vertigo frequently occurred during sexual intercourse, but also during exercises such as jogging and swimming. Examination showed no spontaneous, GEN, or positional nystagmus, but left-beating nystagmus after horizontal head-shaking and during vibratory stimuli applied to either mastoid or brow. PTA documented fluctuating right sensorineural hearing loss especially involving the low-frequency with a pure tone average of 55 dB. The results of bithermal caloric tests, cervical and ocular VEMPs were normal. Brain MRIs taken elsewhere were normal. The patient was diagnosed with MD, and showed no occurrence of vertigo for more than 1 year with medication of 12 mg of betahistine, 60 mg of nimodipine, and 25/25 mg of spironolactone/hydrochlorothiazide per day.

#### Patient 3

A 40~45-year-old previously healthy woman presented with recurrent spontaneous vertigo and ear fullness for 2 months. Two months before presentation, the patient had experienced spontaneous vertigo and ear fullness in the left ear lasting nearly 12 h. After 1 month, the vertigo occurred during sexual intercourse, especially while the patient was experiencing orgasm. It lasted 4 h in association with nausea/vomiting, urination, ear fullness and hearing loss in the left ear. The patient did not show spontaneous nystagmus with or without fixation. Provocative maneuvers including head-shaking, vibration, positional changes, and hyperventilation did not evoke any nystagmus. Bedside HITs were normal in either side. Pure-tone and speech audiometry was normal. MRIs including images for the inner ear and MR angiography did not reveal any responsible lesion. The patient was placed on 48 mg of betahistine and 80 mg of Ginko Biloba per day. The patient did not report further attacks of vertigo during 3 months of follow-up.

#### Patient 4

**Figure 1:**
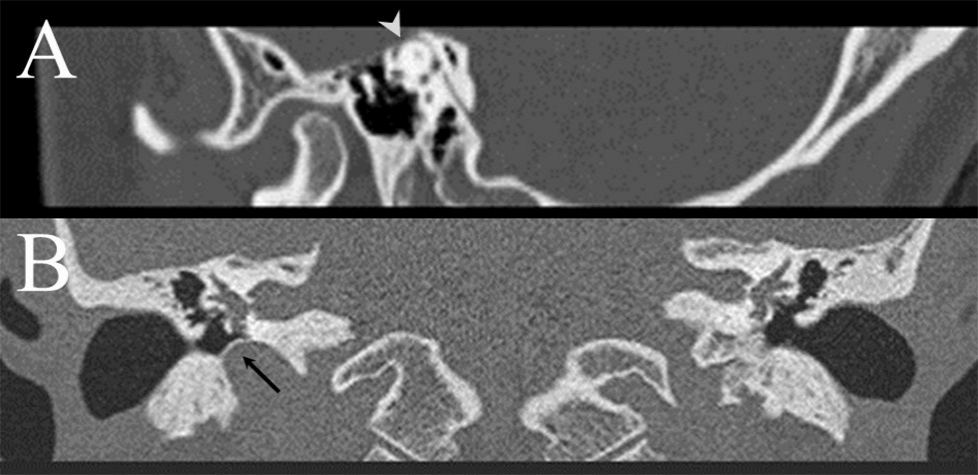
**(A)** Temporal bone CT demonstrates dehiscence of left superior semicircular canal in patient 4 (arrow head). **(B)** Temporal bone CT of patient 5 discloses high position of right jugular bulb with a proximity to the middle ear cavity and vestibular organ, with a thin sigmoid plate covering the roof of the jugular bulb (arrow).

A 30~35-year-old woman presented with recurrent vertigo, tinnitus and autophony for 1 month. The vertigo mostly occurred during sexual intercourse or while listening to loud music. Examination showed no spontaneous, GEN, HSN, or positional nystagmus. However, vibratory stimuli applied to either mastoid or brow evoked downbeat nystagmus with a counter-clockwise (from the patient's view) torsional component while tone burst stimulation of the left ear produced mainly upbeat and clockwise torsional nystagmus. Hyperventilation did not produce any nystagmus. Video HITs were normal for all semicircular canals. PTA showed low-frequency sensorineural hearing loss in the left ear. The threshold of cervical VEMPs was reduced to 65 dB in the left ear, 20 dB lower than that in the right ear. Temporal bone CT disclosed dehiscence of left superior semicircular canal (Figure 1A).

#### Patient 5

A 50~60-year-old man presented with recurrent vertigo during sexual intercourse for 3 years. The vertigo developed exclusively during coitus. The patient denied any vertigo in association with other physical activities, except mild dizzy feeling when he was fasting. The vertigo was mostly spinning for 20–30 min and was associated with nausea and vomiting. The patient also had throbbing headache in the frontal area along with the vertigo, but denied associated diplopia, tinnitus, ear fullness, weakness, or sensory change. Video-oculography showed no spontaneous or GEN. However, he developed left-beating nystagmus after horizontal head-shaking, but without VIN or positional nystagmus. Bedside HITs were normal. The results of bithermal caloric tests, PTA, and ocular and cervical VEMPs were normal. Temporal bone CT and brain MRIs revealed high position of right jugular bulb with a proximity to the vestibular organ, and encroachment on the cochlear and vestibular aqueducts (Figure 1B).

#### Patient 6

A 30~35-year-old woman had suffered from recurrent vertigo for 1 year. The vertigo developed whenever the patient had coitus, especially during the orgasm, and disappeared within 5 min. The vertigo spells were mostly associated with nausea, vomiting, urge of defecation, and hyperacusis. Otherwise, the patient denied any tinnitus or headache. Similar episodes also occurred during other physical activities including swimming or intense exercise. Examination showed no spontaneous or evoked nystagmus. Bedside HITs were normal in all directions. The patient did not have spontaneous, GEN, VIN, or positional nystagmus, but showed right-beating nystagmus after horizontal head-shaking. PTA, rotatory chair, and bithermal caloric tests were normal. No underlying causes were identified in this patient.

#### Patient 7

A 45~50-year-old man presented with recurrent vertigo and tinnitus for 5 years which lasted about 1 h. The attacks had occurred exclusively during sexual intercourse or masturbation, but not during any other physical activities. The patient showed normal findings of bedside and laboratory neurotological evaluation that included video-oculography, PTA, and temporal bone CT.

### Clinical Characteristics

Our patients had suffered from coitus-induced vertigo for 1 month to 6 years (median: 2.5 years). Although the duration of attacks may vary from minutes to 1 h, all patients reported true spinning sensation. In addition, most patients (6/7, 86%, Table 1) reported associated auditory symptom; tinnitus (*n* = 4), ear fullness (*n* = 2), autophony (*n* = 1), hearing impairment (*n* = 1), or hyperacusis (*n* = 1). Four patients reported the vertigo to occur exclusively during sexual intercourse or masturbation while the other three patients also experienced vertigo during other physical activities.

**Table 1:** Clinical findings in the patients.

**Pt**	**Age**	**Diagnosis**	**Clinical presentation**			**Video-oculography and other laboratory findings**				
			**Associated symptoms**	**Duration**	**Other provoking factors**	**SN**	**GEN**	**HSN**	**VIN**	**Other findings**
1	F/65-70	MD, Lt	N/V, tinnitus	<30 min	Increased frequency after estrogen replacement	–	–	–	–	SNHL, Lt (45 dB)
2	M/55-60	MD, Rt	N/V, tinnitus, urge to defecate	20–30 min	Exercise	–	–	4L	4L	SNHL, Rt (50 dB)
3	F/40-45	MD, Lt	N/V, EF, HL, urge to urinate	3–12 h	Not by any other exercises	–	–	–	–	
4	F/30-35	SCD, Lt	EF, tinnitus, autophony	30 min	Listening to music, exercise	–	–	–	6D 3CCW	Tullio phenomenon (+) Decreased cVEMP threshold: 65 dB, Lt SNHL, Lt (30 dB)
5	M/55-60	HJBA, Rt	N/V, headache	20–30 min	–	–	–	8L	–	
6	F/30-35	?	N/V, urge to defecate, hyperacusis	a few min (<5 min)	Exercise (swimming)	–	–	3R	–	
7	M/45-50	?	Tinnitus	1 h	Masturbation, not by any other exercises	–	–	–	–	

*cVEMPs indicates cervical vestibular-evoked myogenic potentials; EF, ear fullness; GEN, gaze-evoked nystagmus; HJBA, high jugular bulb anomaly; HL, hearing loss; HSN, head-shaking nystagmus; HV, hyperventilation; L, left-beating; Lt, left; N/V, nausea/vomiting; MD, Meniere's disease; R, right-beating; Rt, right; SN, spontaneous nystagmus; SNHL, sensorineural hearing loss; VIN, vibration-induced nystagmus*.

None of the patients showed any abnormalities on general physical and neurological examinations. Underlying disorders included MD (*n* = 3, patients 1–3), superior canal dehiscence (*n* = 1, patient 4), and high jugular bulb anomaly (*n* = 1, patient 5), while the remaining two patients were idiopathic (Table 1).

### Neuro-Otological Findings

HSN was observed in three and VIN in two patients. However, none of the patients showed spontaneous, GEN, abnormal HITs, or positional nystagmus. Except patient 4 with Tullio phenomenon from SCD, no one showed positive fistula test or hyperventilation-induced nystagmus.

Other vestibular function tests including bithermal caloric tests (*n* = 4), cervical (*n* = 1) and ocular (*n* = 2) VEMPs, and rotatory chair tests (*n* = 1) were all normal. One patient with SCD showed a decrease of cervical VEMP threshold by more than 20 dB. While brainstem auditory evoked potentials were normal in two patients tested, PTA frequently showed sensorineural hearing loss (3/7, 43%).

## Discussion

Metabolic changes during sexual intercourse include a marked rise in blood pressure, and pulse and respiratory rates. This autonomic arousal may lead to systemic vasocongestion built up during the excitement and plateau phases, and subsequent rapid release of the vascular and muscle tones as a result of orgasm. These systemic changes during the intercourse are known to be mediated by oxytocin (21). In humans, the plasma oxytocin level rises during sexual arousal with a greater increase during the orgasm. Administration of naloxone that blocks the periorgasmic oxytocin release decreases the arousal and orgasm (22). Along with the activated autonomic nervous system, the oxytocin, a potent vasodilator, is regarded to be involved in the central control of blood pressure and systemic and central nervous system vasocongestion (21, 23, 24).

Although the mechanisms of coital vertigo are largely unknown (25), those may be explained by the hormonal changes, especially the rise of oxytocin during the coitus and orgasm. Indeed, two of our patients (patients 3 and 7) reported vertigo to occur exclusively during the orgasm, but not during or after any other physical activities. Also, another patient (patient 1) reported an increase in the frequency of coital vertigo after hormone replacement therapy. By convention, when an acute vertigo spell occurs during sexual intercourse, and if it is monophasic, clinicians mostly suspect a vascular event involving the vertebrobasilar artery. However, our patients showed that peripheral vestibulopathy should be suspected in recurrent coital vertigo.

Otherwise, physiologic changes occurring in association with the exertion during sexual intercourse may play a role in generating coital vertigo. It is noteworthy that nearly a half of the patients reported their vertigo also to occur during exercises requiring physical strains other than sexual intercourse. According to this observation, the vertigo may be just an epiphenomenon of coitus and orgasm rather than a direct effect of those. Indeed, it is common to encounter individuals with vestibular deficits who report exacerbation of their symptoms during physical or emotional stress (26). Otherwise, exertion itself can be the primary cause of vertigo in some patients (27). Accordingly, some patients with exertional dizziness/vertigo reported reproduction of their symptoms during hyperventilation in the absence of any nystagmus (27). Indeed, more than a half (67%) of exertional dizziness replicates symptoms during the tilt table or lactation test, and it is better explained by autonomic dysregulation rather than by primary neuro-otologic etiologies (27).

The balance of endolymphatic pressure is dependent on ICP that is transmitted to the peri- and endolymph via the cochlear aqueduct and endolymphatic sac (28). The endolymphatic sac and cochlear aqueduct are known to modulate the pressure transferred to the labyrinth (29). Indeed, the disorders associated with an ICP changes [e.g., post-lumbar puncture headache (30), spontaneous intracranial hypotension (31), increased ICP headache (32)] usually accompany auditory symptoms. Therefore, MD or perilymph fistula including SCD, which involves the pressure buffering system, may develop dizziness/vertigo in associated with ICP changes (33). Endolymphatic hydrops hinders labyrinthine adjustments to ICP changes, leading to an alteration in the labyrinthine microvascular blood flow, and this, in turn, can cause vertigo, tinnitus and hearing loss (34). In addition, the frequent association of auditory symptoms in our patients also support this ICP hypothesis as the mechanism of coital dizziness/vertigo. Otherwise, as oxytocin increases cerebral blood flow by vasoconstrictory effect on small cerebral resistance vessels (35), vasoconstriction in the inner ear may trigger dizziness/vertigo in our patients. Indeed, autoregulation of cochlear blood flow is impaired in endolymphatic hydrops and induction of Meniere attacks requires both hydrops and inner ear perfusion (36). Likewise, sudden deafness may occur in relation to sexual intercourse, probably due to impaired cochlear microcirculation (37).

Of interest, one of our patients showed high jugular bulb anomaly. High position of the jugular bulb is a well-recognized anatomical variation and is observe in 6~10% of the temporal bones (38, 39). It may present cochleovestibular symptoms due to transmission of the pressure from the high jugular bulb to the inner ear structures responsible for endolymph resorption (38, 40). Indeed, high jugular bulb was found in 57% of patients with definite MD, and encroached upon the cochlear and vestibular aqueducts in ~35–40% of them (41). Also, surgical correction of this high jugular bulb resulted in resolution of tinnitus (7/13, 54%) and vertigo (12/13, 92%) in patients with MD (38). Based on these, the coital vertigo in our patient with high jugular bulb anomaly may be explained by the unopposed transmission of the ICP to the inner ear.

Previously, coitus-related headache has received more attention than coitus-related vertigo, and had diverse causes from benign conditions such as benign coital headache to malignant ones including subarachnoid hemorrhage or embolic stroke (4, 9). These headaches may occur before or during orgasm and may be classified into the pre-orgasm and orgasm subtypes (42). Patients with coital headache are known to have comorbid migraine, benign exertional headache, cluster headache, and tension-type headache (2). The pathophysiology of coital headache has not been fully understood, but most authors have assumed that the orgasmic subtype may be due to dysregulation of the arterial blood pressure with dilated intracranial vessels and increased ICP (43, 44). Similar mechanism may play a role in generating coital vertigo, but we assume that isolated ICP increment alone would not be sufficient to evoke vertigo, and the buffering system, which refrain the ICP from being directly transferred to the peripheral vestibular organs, should have been affected.

In addition to MD, SCD or high jugular bulb, the list of differential diagnoses for vertigo induced during coitus should include benign paroxysmal positional vertigo (BPPV), Chiari malformation, vertebrobasilar insufficiency, migraine, subarachnoid hemorrhage, and reversible vasoconstriction syndrome even though several of those mostly accompany headaches. Given the underlying disorders detected in our patients with vertigo induced during coitus, neurotologic evaluation should take place prior to ordering tests to investigate intracranial pathologies, especially for those temporal bone CT appears more sensitive than MRIs.

Unlike in other vestibular disorders characterized by triggered vertigo or nystagmus (ex. BPPV or perilymph fistula), the causal relationship between coitus and vertigo attacks could not be established in our study and would be hardly possible in most cases with vertigo induced during coitus. Thus, from a scientific point of view, the diagnosis remains a possibility. Although we were able to find only seven cases from the data comprising more than 3,990 patients with recurrent dizziness/vertigo (0.2%) (25, 45), the true incidence of coital vertigo remains unknown. Its prevalence should be much higher since the detection was mostly based on the patients' voluntary report. Given that sexual activities still belong mostly to the intimate domain, under-report of coital vertigo is well-presumed. Every aspect of this potentially discouraging phenomenon requires further exploration.

## Author Contributions

S-UL analyzed and interpreted the data, and wrote the manuscript. H-JK, J-WK, and J-YC analyzed and interpreted the data, and revised the manuscript. J-SK designed and conceptualized the study, interpreted the data, and revised the manuscript.

### Conflict of Interest Statement

J-WK serves on the editorial boards of the Journal of Vestibular Research and Auris Nasus Larynx. J-SK serves as an associate editor of Frontiers in Neuro-otology and on the editorial boards of the Journal of Clinical Neurology, Frontiers in Neuro-ophthalmology, Journal of Neuro-ophthalmology, Journal of Vestibular Research, Journal of Neurology, and Medicine. The remaining authors declare that the research was conducted in the absence of any commercial or financial relationships that could be construed as a potential conflict of interest.
